# Supplementary figures and images for: RNA binding protein FXR1-miR301a-3p axis contributes to p21WAF1 degradation in oral cancer
Source: PLoS Genet. 2020 Jan 15;16(1):e1008580. doi: 10.1371/journal.pgen.1008580 (PMC6986764; doi:10.1371/journal.pgen.1008580)

Fig. S1

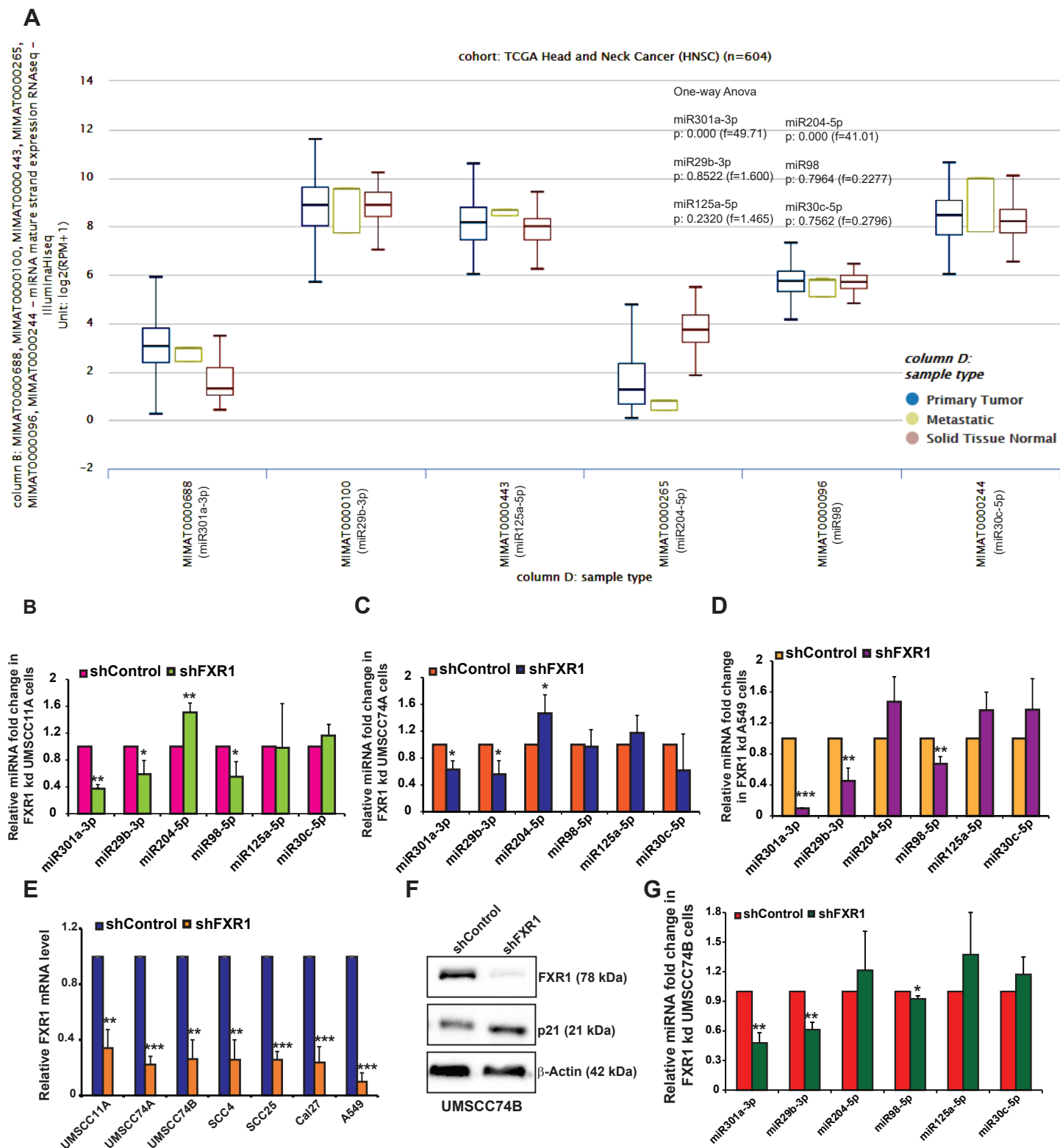

Supplement: S1 Fig — (A) Box plot with one-way ANOVA of differential miRNA expression in the 604 patients obtained from the TCGA HNSCC database. Blue: primary tumor (n-483), green: metastatic tumor (n = 2), and brown: solid tissue normal (n = 44). X-axis signifies the sample type where y-axis plots the log-RPM (reads per million)-values of the differentially expressed miRNAs in HNSCC. (B) qRT-PCR of altered miRNAs in FXR1 KD UMSCC11A cells. RNU6 served as an endogenous control. (C) qRT-PCR of altered miRNAs in FXR1 KD UMSCC74A cells. RNU6 served as an endogenous control. (D) qRT-PCR of altered miRNAs in FXR1 KD A549 cells. RNU6 served as an endogenous control. (E) qRT-PCR is showing the KD efficiency FXR1 in shFXR1 (used in Fig 1) treated cells used for miRNA analyses. Actin and GAPDH served as endogenous controls. (F) Western blot showing the KD efficiency of FXR1 by shRNA (TRCN0000158932) compared to a scrambled shRNA where β-Actin serves as a loading control. (G) qRT-PCR of altered miRNAs in FXR1 KD (TRCN0000158932) UMSCC74B cells. RNU6 served as an endogenous control. Data from B-D and F-G represent the mean of n = 3 experiments. Statistical significance (p-value): *<0.05; **<0.005; ***<0.0005. (PDF) [file pgen.1008580.s001.pdf]

Figure S2

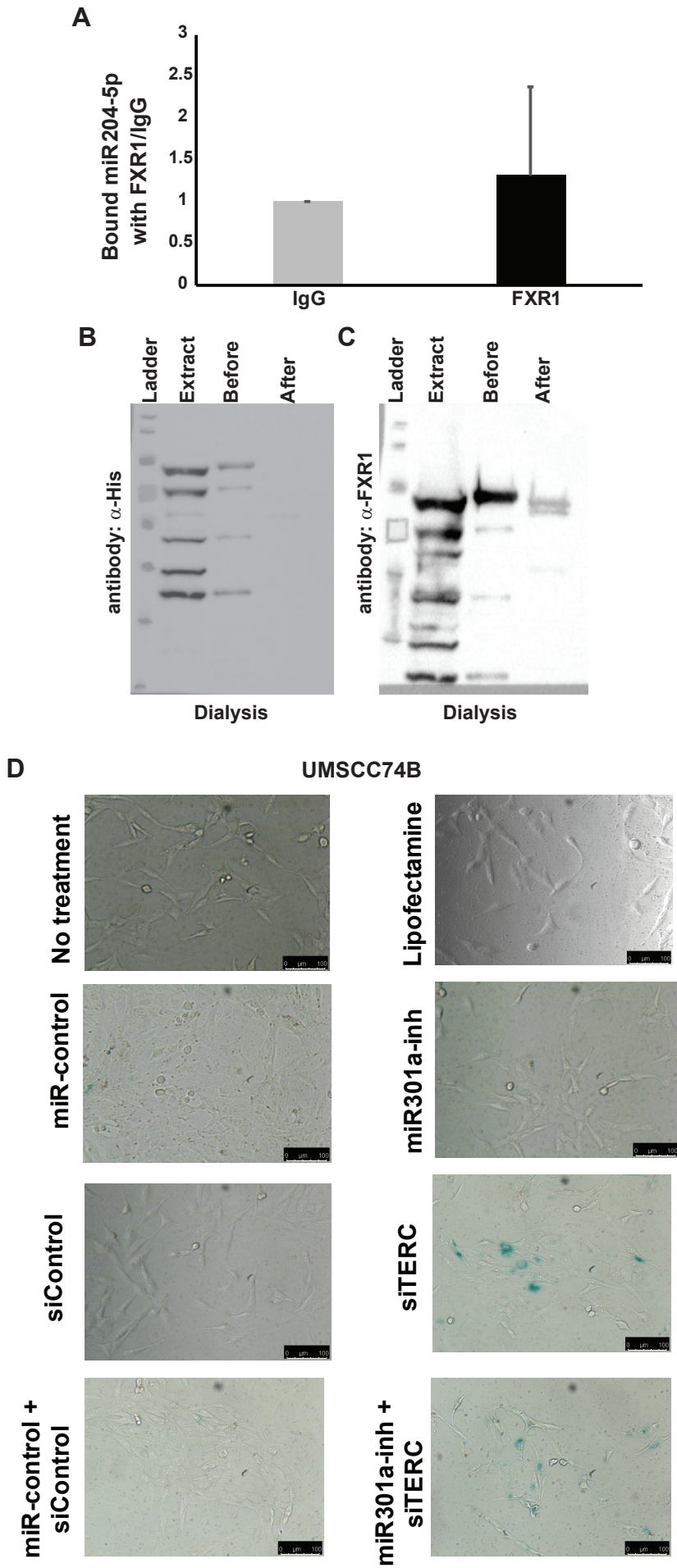

Supplement: S2 Fig — (A) RNA-immunoprecipitation shows miRNA204-5p does not bind to FXR1 in UMSCC74B cells compared to control mouse IgG. Both ACTIN and RPS18 served as endogenous controls. (B) Western blot analyses showing recombinant FXR1 protein expression before and after dialysis with the anti-His-tag antibody. (C) Western blot analyses showing recombinant FXR1 protein expression before and after dialysis with the anti-FXR1 antibody. (D) Beta-galactosidase assay showing, like the previous observation [34], instead of the miRNA alone, both miR301a-3p and TERC downregulation can induce senescence in UMSCC74B cells. (PDF) [file pgen.1008580.s002.pdf]

Fig.S3

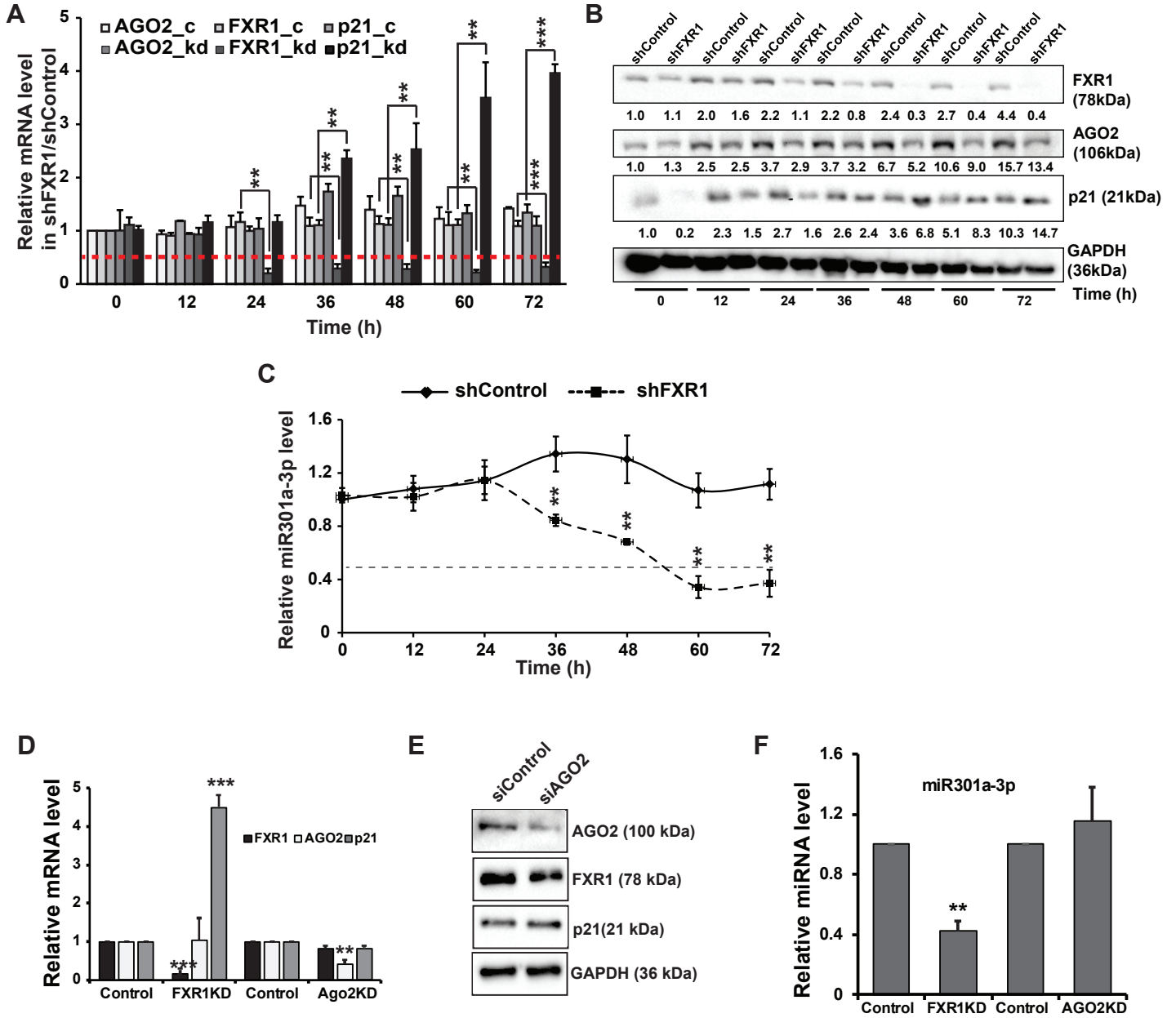

Supplement: S3 Fig — (A) qRT-PCR assay of FXR1 KD UMSCC11A cells showing significant down- and up-regulation of FXR1 and p21, respectively, compared to control. AGO2 did not show any change after FXR1 KD. Both ACTIN and RPS18 served as endogenous controls. (B) Western blot analyses of FXR1, p21, and AGO2 from UMSCC11A cells collected at different time points after FXR1 KD. GAPDH serves as a loading control. (C) qRT-PCR assay of FXR1 KD UMSCC11A cells showing significant miR301a-3p decay from 48 hrs compared to control. Cells were collected at the designated time points after shRNA transduction. RNU6 served as an endogenous control. (D) qRT-PCR assay of FXR1 and AGO2 KD UMSCC74B cells. Unlike FXR1 KD cells, FXR1 and p21 did not show any biologically relevant changes after AGO2 KD. Both ACTIN and RPS18 served as endogenous controls. (E) Western blot analyses of FXR1, p21, and AGO2 from UMSCC74B cells after AGO2 KD. GAPDH serves as a loading control. (F) qRT-PCR assay of AGO2 KD UMSCC74B cells showing no significant regulation of miR301a-3p compared to control at 72hrs of transduction. RNU6 served as an endogenous control. Data here represents the mean of n = 3 experiments. Statistical significance (p-value): *<0.05; **<0.005; ***<0.0005. (PDF) [file pgen.1008580.s003.pdf]

Fig. S4

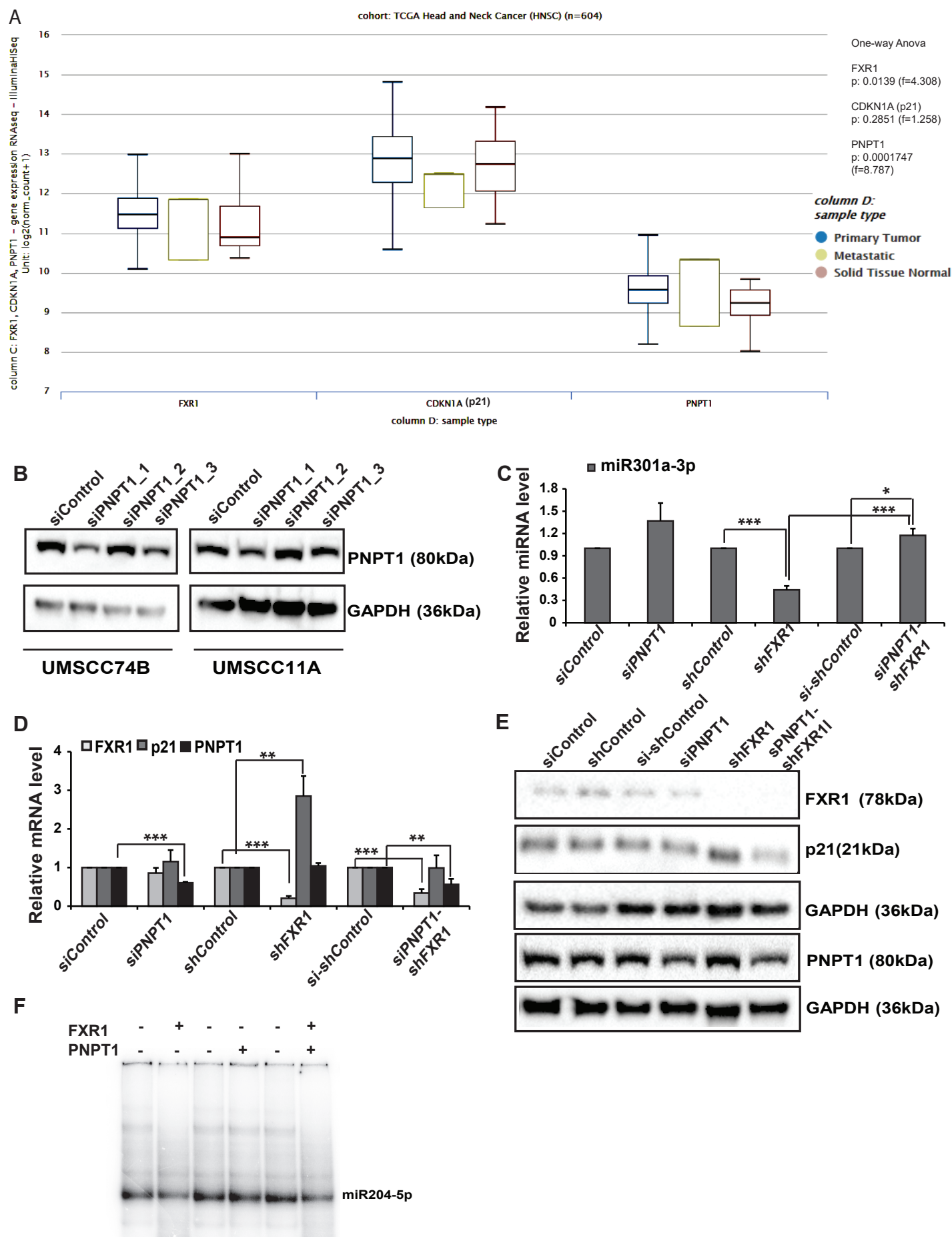

Supplement: S4 Fig — (A) Box plot with one-way ANOVA of differential miRNA expression in the 604 patients obtained from the TCGA HNSCC database. Blue: primary tumor (n = 520), green: metastatic tumor (n = 2), and brown: solid tissue normal (n = 44). X-axis signifies the sample type where y-axis plots the log-RPM (reads per million)-values of the differentially expressed miRNAs in HNSCC. (B) Western blot to test the KD of PNPT1 in UMSCC74B and UMSCC11A cells with three siRNAs at 50nM. GAPDH serves as a loading control. (C) qRT-PCR analyses to test the expression of miR301a-3p in UMSCC11A cells under individual and double KD of FXR1 and PNPT1. RNU6 served as an endogenous control. (D) qRT-PCR analysis of FXR1, p21, and PNPT1 in UMSCC11A cells under individual and double KD of FXR1 and PNPT1. Both ACTIN and RPS18 served as endogenous controls. (E) Western blot analysis of FXR1, p21, and PNPT1 in UMSCC11A cells under individual and double KD of FXR1 and PNPT1. GAPDH serves as an endogenous control. (F) EMSA shows that both rFXR1 and rPNPT1 proteins are unable to bind and degrade, respectively, the in vitro transcribed miR204-5p. Data here represent the mean of n = 3 experiments. Statistical significance (p-value): *<0.05; **<0.005; ***<0.0005. (PDF) [file pgen.1008580.s004.pdf]

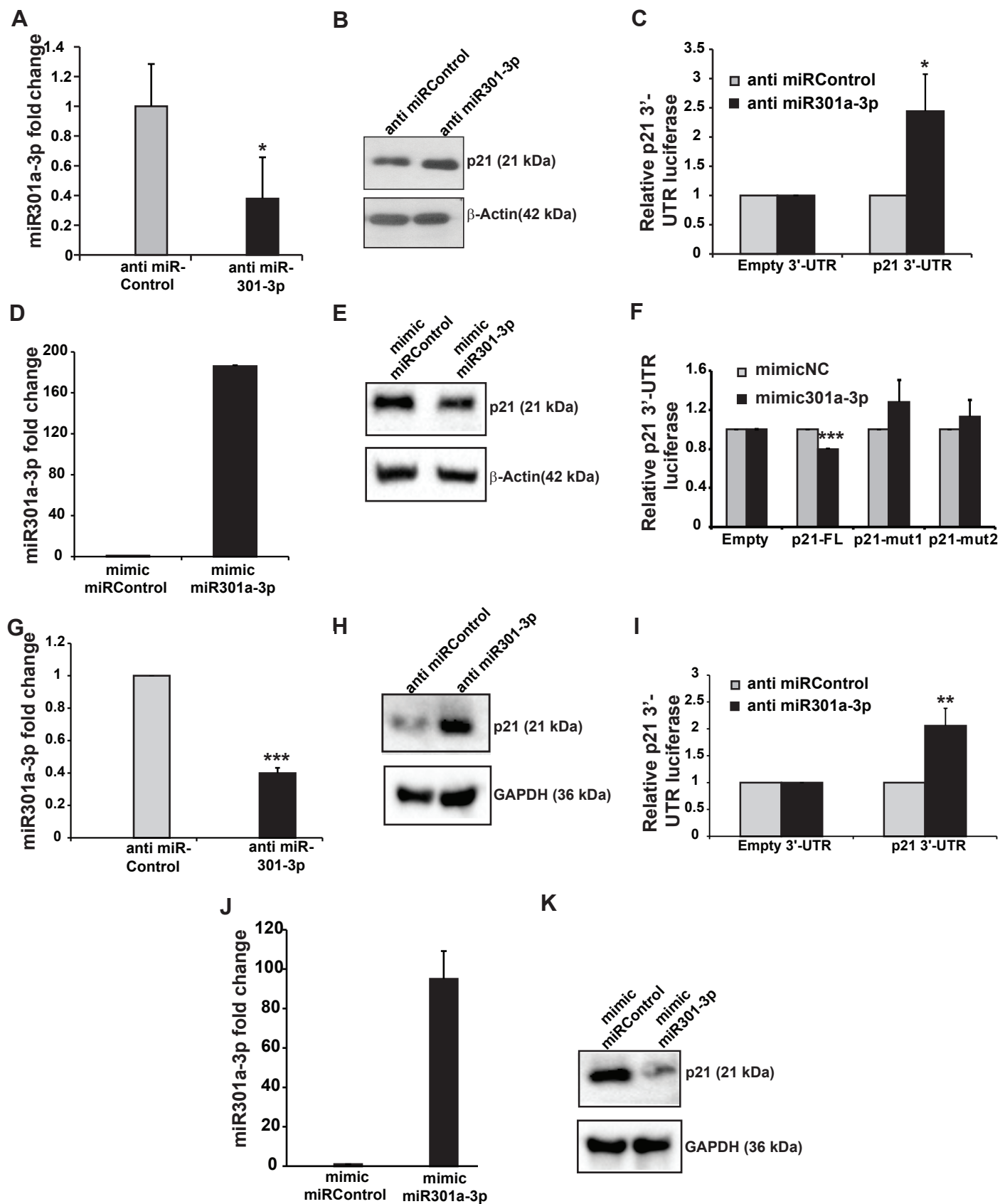

Supplement: S5 Fig — (A) qRT-PCR analyses to test the expression of miR301a-3p in UMSCC74A cells treated with miRNA inhibitor with scrambled control. RNU6 served as an endogenous control. (B) p21 protein is up-regulated in miR301a-3p inhibitor transfected UMSCC74A cells. β-Actin serves as a loading control. (C) p21 3’UTR luciferase activity is significantly up-regulated in the presence of miR301a-3p inhibitor in UMSCC74A cells compared to the scrambled control transfected cells. Forty-eight hours after transfection of UMSCC74A cells with miRNA control and 301a-3p inhibitor along with empty 3’-UTR luciferase plasmid and wild type p21 3′-UTR, the lysates were analyzed for luciferase activity using a luminometer. The empty 3’UTR luciferase plasmid served as a transfection and loading control. Values are the means ± SD from three independent experiments by using an unpaired two-sample t-test. (D) Expression of miR301a-3p in UMSCC74A cells treated with miRNA mimics. RNU6 served as an endogenous control. (E) p21 protein is down-regulated in miR301a-3p mimic treated UMSCC74A cells. β-Actin serves as a loading control. (F) p21 3’-UTR (full-length wild type and mutated miRNA binding sites) luciferase activity with an expression of miR301a-3p mimic in UMSCC74A cells. In the presence of miR301a-3p mimic, the p21 3’-UTR luciferase activity significantly reduces whereas the mutants show a highly significant up-regulation. Experiments were performed as described in (C). (G) qRT-PCR analyses to test the expression of miR301a-3p in A549 cells treated with miRNA inhibitor with scrambled control. RNU6 served as an endogenous control. (H) p21 protein is up-regulated in miR301a-3p inhibitor transfected A549 cells. GAPDH serves as a loading control. (I) p21 3’UTR luciferase activity is significantly up-regulated in the presence of miR301a-3p inhibitor in A549 cells compared to the scrambled control transfected cells. Experiments were performed as described in (C). The empty 3’UTR luciferase plasmid served a [file pgen.1008580.s005.pdf]
