# Supplementary material for: RNA binding protein FXR1-miR301a-3p axis contributes to p21WAF1 degradation in oral cancer
Source: PLoS Genet. 2020 Jan 15;16(1):e1008580. doi: 10.1371/journal.pgen.1008580 (PMC6986764; doi:10.1371/journal.pgen.1008580)
Supplement: S1 Table — (DOCX) [file pgen.1008580.s006.docx]

| **Primer** | **Sequence (5’-3’)** |
| --- | --- |
| FXR1-F | CCCTAATTACACCTCCGGTTATG |
| FXR1-R | TCTCCTGCCAATGACCAATC |
| AGO2-F | TACAAGGCACAGCCAGTAATC |
| AGO2-R | CAGTGCGTTATCTCCACCTTTA |
| pET28aFXR1-F | TCGCGGATCCGAATTCATGGCGGAGCTGACGGTG |
| pET28aFXR1-R | GTGCGGCCGCAAGCTTTTATGAAACACCATTCAGG |
| P21-F | CGGAACAAGGAGTCAGACATT |
| P21-R | AGTGCCAGGAAAGACAACTAC |
| pLS_p213’UTR-F1 | CTAATCTAGAGCTAGCCACAGCCTAGGGCTGAGCTG |
| pLS_p213’UTR-R | GCGGCCGGCCCTCGAGAAGCACTTCAGTGCCTCC |
| pLS_p213’UTRmut1-F | CATGGCCCCTCTGACCTCGTGTGGGGAGCCCGTCTCAG |
| pLS_p213’UTRmut1-R | CTGAGACGGGCTCCCCACACGAGGTCAGAGGGGCCATG |
| pLS_p213’UTRmut2-F | CATCCCTCCCCAGTTCATACGTGTTTGATTAGCAGCGGAAC |
| pLS_p213’UTRmut2-R | GTTCCGCTGCTAATCAAACACGTATGAACTGGGGAGGGATG |
| miR301a-3p-R (DNA probe) | GCTTTGACAATACTATTGCACTG |
| snRU6-R (DNA probe) | TATGTGCTGCCGAAGCGAGCAC |
| miR301a-3p (sigma) for EMSA | CAGUGCAAUAGUAUUGUCAAAGC |
| miR204-5p (sigma) for EMSA | UUCCCUUUGUCAUCCUAUGCCU |
| dSi-AGO2-F | rGrArArCrUrArArGrGrArGrCrArGrUrGrGrCrArGrArArGGT |
| dSi-AGO2-R | rArCrCrUrUrCrUrGrCrCrArCrUrGrCrUrCrCrUrUrArGrUrUrCrArG |
| PNPT1-F | GACTGCGGTACTGCTTCATAA |
| PNPT-R | CTGGGTCACGTCCAAAGTATT |
| XRN1-F | CGACTCCCGTTTCTCCAATATC |
| XRN1-R | CTTCTCCAGTGCAGCCATAATA |
| XRN2-F | CTCCTGGTGAAGGAGAACATAAA |
| XRN2-R | TCAGCATCTGCTCCACATAAA |
| SKA2-F | GGAGAGGGAAAGCAGGTATTG |
| SKA2-R | GTGATCTGCACAGGGTCATAG |
| GAPDH-F | GGTGGTCTCCTCTGACTTCAACA |
| GAPDH-R | GTTGCTGTAGCCAAATTCGTTGT |
| Beta Actin-F | GGACCTGACTGACTACCTCAT |
| Beta Actin-R | CGTAGCACAGCTTCTCCTTAAT |
| RPS18-F | CTTTGCCATCACTGCCATTAAG |
| RPS18-R | ATCACACGTTCCACCTCATC |

**S1 Table.** Primers used in the study.
