# Supplementary material for: RNA binding protein FXR1-miR301a-3p axis contributes to p21WAF1 degradation in oral cancer
Source: PLoS Genet. 2020 Jan 15;16(1):e1008580. doi: 10.1371/journal.pgen.1008580 (PMC6986764; doi:10.1371/journal.pgen.1008580)
Supplement: S2 Table — (DOCX) [file pgen.1008580.s007.docx]

**S2 Table.** miRNA primers/probes used in the study.

| **miRNA** | **Assay ID#** |
| --- | --- |
| U6 snRNA | 001973 |
| hsa-miR-301a-3p | 000528 |
| hsa-miR-98-5p | 000577 |
| hsa-miR-125a-5p | 002198 |
| hsa-miR-204-5p-Human | 000508 |
| hsa-miR-30c-5p-Human | 000419 |
| hsa-miR-29b-3p-Human | 000413 |
| TQMN Noncoding RNA Assay for pre-miR301a | Hs04231543_s1 |
| TQMN Noncoding RNA Assay for control 18S rRNA | HS03928990_g1 |
