# Supplementary material for: RNA binding protein FXR1-miR301a-3p axis contributes to p21WAF1 degradation in oral cancer
Source: PLoS Genet. 2020 Jan 15;16(1):e1008580. doi: 10.1371/journal.pgen.1008580 (PMC6986764; doi:10.1371/journal.pgen.1008580)
Supplement: Attachment — Submitted filename: Rebuttal letter.docx [file pgen.1008580.s008.docx]

Dear Editor,

We would like to thank the reviewers for the helpful comments and suggestions. The article has been revised, keeping the comments into consideration. A summary of the same has been provided below, addressing the individual concerns of the reviewers. It is hoped that the explanations and new results offer satisfactory answers to the issues raised. We would be glad to see the manuscript in final acceptance format after completion of this review process. We have addressed all the concerns raised by the reviewers (answers in red) in the past review process and hoping to satisfy the reviewers. Thanking you.

Sincerely,

Viswanathan Palanisamy, PhD

Reviewer #1: In this manuscript, the authors reported that a RNA-binding protein FXR1 control miR301a-3p stability by directly binding to this miRNA and prevent its degradation by PNPT1 in HNSCC. As miR301a targets p21, thereby FXR1 promote the growth and proliferation of HNSCC. The report does contain some innovative findings. The manuscript is well written and all of data are beautifully presented. However, all of data were collected on the cultured cell lines. No data show there is functional relevance in vivo. The reviewer think the report is still preliminary and need to add some in vivo observation.

As suggested by the reviewer, we have now performed nude mice xenograft experiment (Fig.6G) with IPTG inducible FXR1 knockdown UMSCC74B cell lines. 50% of each of male and female mice (12) were divided and injected with UMSCC74B cells stable expressing IPTG inducible shControl (left) and shFXR1 (right) on each of the flanks. 50% of the population was treated with IPTG in the drinking water the following day of injection. And the rest 50% was treated after the first nodule was observed. As can be seen from the result in Fig6.G the first group failed to grow any tumor in FXR1 knockdown side whereas the second batch, the male mice had the nodule on the shFXR1 injected side which did not grow up to the control. We did not observe any nodule the second batch of female mice after dissection. We have also provided the protein (FXR1 and p21) and miR301a-3p data from the 3 small tumors (M#4-6) compared to control.

Reviewer #2: The manuscript “RNA binding protein FXR1-miR301a-3p axis contributes to p21WAF1 degradation in oral cancer” provides a compelling case that FXR1 protects miR301a-3p from degradation from exonuclease PNPT1 which allows the miR to destabilize p21, forming a novel axis of regulation in oral cancer cells. Overall, I find this manuscript to be of great interest to the fields of both RBP-miR interactions and RNA regulation of cancer biology.

Major Critiques:
1. Figure 2A needs a negative control gene in addition to the IgG control shown for FXR1-miR 301a-3p interaction by pulldown.

We have now added a negative control data in FigS2A. miR204-5p did not show any significant binding FXR1 protein by RNA-immunoprecipitation.
2. Figure 2 shows an EMSA gel shift experiment, but the figure would be more convincing and enhanced using a negative control probe to demonstrate specificity in FXR1 binding in vitro.

We have now added miR204-5p data there in FigS4F. In vitro transcribed miR204-5p (Sigma) failed to bind to FXR1 protein or did not get degraded by PNPT1.
3. Figure 4E again would be enhanced by a negative control probe especially due to degradation of miR301a-3p probe by PNPT1 to again show specificity of the interaction and degradation.

Kindly see the previous reply.
4. Although it is clear FXR1 binds miR301a-3p, can you rule out that FXR1 directly alters PNPT1 to directly or indirectly prevent miR degradation?

FXR1 knockdown did not have any significant effect on PNPT1 RNA or protein as the existing data (Fig 4 and S4) show. Hence, we omitted further analysis of PNPT1. Our ongoing studies now aim at understanding the mechanisms of PNPT1 and FXR1 in RNA decay studies in HNSCC.

5. The overall interaction axis between FXR1-miR301a-3p-p21 suggests effects on cellular senescence and proliferation, and experiments measuring changes in the cell would strengthen the paper demonstrating tangible effects of this interaction in cancer cells. These cellular experiments should be performed manipulating participating components of the novel axis to determine effects on cellular function.

Our previous Plos Genetics publication (<https://www.ncbi.nlm.nih.gov/pubmed/27606879>) showed that FXR1 regulates both p21 and TERC RNA to bypass senescence. We have now added data in the supplementary section (Fig. S2D) showing how controlling both miR301a-3p and TERC RNA has affected cellular senescence in UMSCC74B cells.

Minor Critiques:
1. The paper introduces and shows data involving the FXR1-miR29b-3p interaction but fails to explain why this was not continued.

The goal of this paper was to understand if FXR1 involves miRNA to degrade p21 mRNA. miR29b-3p did not show a binding site on p21from Targetscan, so we decided not to pursue the miRNA after validation for this specific manuscript. We are separately working on miR29b-3p for another project. However, we have clarified that in the result section for Fig1.
2. Discussion points: Does miR301a-3p target any other cancer or senescence related transcripts other than p21 based on the target sequence? Further, does FXR1 bind p21 directly and regulate the transcript by RNA stability, and how that might affect the role of FXR1 in the axis identified.

We have now added that to the discussion.

Reviewer #3: Majumder and Palanisamy describe a novel and very relevant regulatory model involving miR-301a and the RNA binding protein FXR1. The data is convincing and supports their model. However, there are a few weak points and further analysis should be done to expand their conclusions and increase the value of their article.

Major points
-Changes in transcription levels could interfere with results. Transcription should be shut down, then they can properly evaluate the impact of FXR1 on miR-301a stability.

We have now performed Actinomycin D experiment to show (Fig 3D) how FXR1 knockdown completely abolishes miR301a-3p level by northern blot when compared to Actinomycin D/DMSO treatment.
-In Figure 2C, it would be good to have a negative control with a mutated miR-310a.

We thank the reviewer for this comment. We have now added miR204-5p [failed to bind to FXR1 in vivo by RNA-IP (FigS2A)] data there in FigS4F. In vitro transcribed miR204-5p (Sigma) failed to bind to FXR1 protein or did not get degraded by PNPT1.
-There is probably CLIP data available for FXR1. The authors should check if miR-310a is described as a binding target and determine the sequence that is recognized.

We have checked the CLIP data however, we could not to find substantial miRNA binding to FXR1 which can be difference between two different cancer lines as the CLIP was done on HepG2 cells.
-Binding of PNPT1 to miR-301a is not shown.

The data was already there. Kindly check Fig4E lanes 3 and 6.

-In fact, their model can have two components and they have to check that. It looks like that binding and function of FXR1 to p21 transcript is influenced by miR301 binding and vice versa. Are miR301a and FXR1 binding sites in p21 3'UTR close by? They can check for instance using luciferase constructs if mutation of miR301a binding site in p21 3'UTR abolishes FXR1 binding and regulation. Similarly, they can mutate FXR1 site and determine if miR301a regulation is affected.

Thank you for this comment. Part of this was already shown in our previously published paper (<https://www.ncbi.nlm.nih.gov/pubmed/27606879>) where we show the FXR1 binding sites on p21. Unfortunately, FXR1 preferentially binds to G quartet sequences and human p21 has G quartet spanned along the 3’UTR. So, we showed only miRNA binding sites (by mutation) in this manuscript which falls within FXR1 binding region.

Minor points:
-The authors should check the expression correlation between FXR1 and miR-301a in TCGA head and neck cancer and impact on survival.

Currently, XENA does not provide a survival plot for miRNA and for miRNA-protein combination. However, we looked in ENCOREI (starbase v3.0), that uses data from TCGA, and found that FXR1 and miR301a-3p are show a significant co-expression in most cancers including HNSCC (497 patients). As we do not have the survival plot yet, we did not use this information in the manuscript.
-The introduction is somehow confusing and goes back and forth talking about miRNAs and RBPs.

We have rearranged a few things in the introduction.
-The authors should try to go beyond their model. This could really increase the value of their article. miRNAs and RBPs can be part of regulatory networks, for instance Musashi1 and miR-137 (31004009). Using available data and predictions, they could check if FXR1 and mir301a co-regulate other target genes.

Currently, we are testing FXR1 for targets using different existing datasets. However, different cancers can have different targets for the same protein/miRNA axis. Therefore, we are also trying PARCLIP and simple RNAseq in HNSCC cell lines.
